# New Aspects of Degradation in Silicone Rubber under UVA and UVB Irradiation: A Gas Chromatography–Mass Spectrometry Study

**DOI:** 10.3390/polym13132215

**Published:** 2021-07-05

**Authors:** Zheng Wang, Libing Qian, Xiangyang Peng, Zhen Huang, Yue Yang, Chunqing He, Pengfei Fang

**Affiliations:** 1 Guangdong Key Laboratory of Electric Power Equipment Reliability, Electric Power Research Institute of Guangdong Power Grid Co., Ltd., Guangzhou 510080, China; williamwang@whu.edu.cn (Z.W.); xjtuhuangzhen@126.com (Z.H.); 2 Hubei Nuclear-Solid Physics Key Laboratory and Department of Physics, Wuhan University, Wuhan 430072, China; 2017102020009@whu.edu.cn (L.Q.); yangyue@whu.edu.cn (Y.Y.)

**Keywords:** silicone rubber, gas chromatography-mass spectrometry, positron annihilation lifetime spectroscopy, UVA/UVB irradiation, silicone oligomers

## Abstract

In this paper, gas chromatography–mass spectrometry (GC–MS) and positron annihilation lifetime spectroscopy (PALS) were used to probe the changes of oligomers and the polydimethylsiloxane (PDMS) network in silicone rubber, after different durations of UVA/UVB irradiation. At the early stage (<300 h) of UVA/UVB irradiation, the concentration of D${}_{4}$-D${}_{9}$ decreases. The o-Ps intensity of the extracted silicone rubber increases in the stage after UVB irradiation. These results indicate the crosslinking of oligomers into the PDMS network. After a long duration (>300 h) of UVB irradiation, D${}_{4}$ was generated and the lifetime of $\tau_{3}$ also increased, indicating the rupture of the Si-O bond in the PDMS network. These two aging processes were termed the post curing process and the chain session process. The new finding was that UVA could only induce the post curing process; UVB causes the rupture of the chemical bond in silicone rubber. Photons of UVB could break the C-H bond, and then trigger the backbiting decomposition of PDMS, breaking the Si-O bond, while the photons of UVA cannot. The fact that D${}_{4}$ was generated after UVB irradiation can be used to evaluate the UVB stability of silicone rubber in the future.

## 1. Introduction

In recent years, polydimethylsiloxane (PDMS) based composite has had a myriad of applications [1,2,3,4,5] such as high voltage external insulation, microfluidic devices and soft lithography, and is promising as an insulation material for superconducting cable, and so forth. In the area of insulation especially, compared with the traditional inorganic insulation materials, PDMS based silicone rubber has advantages such as a lower weight, better hydrophobicity, and unique hydrophobicity recovery [6,7,8]. In the running environment of an insulation device, its surface might be polluted by dust and salt. On encountering wet weather conditions, the polluted surface will form a continuous water film, which is conductive because of the dissolution of salt. As a result, the leakage current and dry-band arcing will develop on an insulation device, finally triggering flashovers on a transmission line. Flashover induced trip is a disaster for the quality of electricity transported in a power grid. The hydrophobicity property of silicone rubber can hinder the formation of water films on its surface, and thus greatly reduce the possibility of flashovers. However, as a kind of polymer, silicone rubber can age and lose its surface hydrophobicity. The amazing thing is that, while the surface hydrophobicity of a silicone rubber can be lost, it can regain this property gradually after a subsequent period of rest. The mechanism of this unique hydrophobicity recovery of silicone rubber has already been intensively studied. Researchers have reached the conclusion that the diffusion of the low molecular weighed (LMW) siloxanes from the bulk to the surface of a silicone rubber is the main mechanism of the hydrophobicity recovery process [9,10]. Thus, characterizing the LMW siloxanes in silicone rubber is vital for evaluating the long time running behavior of a silicone rubber made insulation device.

Existing studies have focused on the variations of LMW siloxanes in silicone rubber treated by aging factors in its typical conditions of use, such as corona discharge, biological environment, and so on. The effect of corona discharge has already been studied by Hillborg et al. [11], and they found that mainly cyclic silicone oligomers of D${}_{4}$–D${}_{9}$ are generated on the surface of silicone rubber after corona aging. Kaali el al. [12] conducted a biodegradation of silicone rubber, and they found that human patient exposure would induce the generation of linear PDMS oligomers in silicone rubber. For a long time, it is has been thought that PDMS is stable in UV sunlight, especially in the UVA wavelength range (300∼380 nm) [13,14,15]. However, for some sunlight rich areas, such as the Yunnan province in China, the aging effect of UVB (280∼340 nm) should be considered. Because the atmosphere is rather thin in plateau places, even UVB can reach the Earth due to the much weaker reflection effect of the atmosphere in these areas. In our previous study, UVB induced aging in silicone rubber was studied with the slow positron beam method, a technique used for depth profile analysis. It was found that an inorganic silica-like layer is formed on the sample’s surface, and the chain scission of PDMS does occur in silicone rubber [16]. In order to acquire a deeper understanding of this UVB induced degradation in PDMS, gas chromatography–mass spectrometry (GC–MS) was used to probe the LMW siloxanes’ variations in silicone rubber.

In this paper, the silicone rubber was irradiated by lamps of sunlight UVA-340 nm and UVB-313 nm, respectively. The LMW siloxanes in the silicone rubber were extracted by a soxhlet extraction process, and the extracts were analyzed by GC–MS. An external standard method was used for further quantitative analysis using GC. In order to study the crosslinking state of the PDMS network, positron annihilation lifetime spectroscopy (PALS) was also used. Based on the obtained results, we found that the Si-O bond of PDMS ruptured after UVB irradiation, and a possible aging mechanism of this degradation process was finally proposed.

## 2. Experiments

### 2.1. Materials Preparation

High-temperature vulcanized silicone rubber was studied. The raw materials used to manufacture the silicone rubber are listed in Table 1. The methyl-teinated PDMS was purchased from Cix Xin Rui Chemical Co., Ltd. (Cixi, China). The molecular weight of the PDMS was 45∼85 × 10${}^{4}$ g/mol. The hydride-functional silicone oils were α,ω-dihydroxyl polydimethylsiloxane, purchased from Hoshine Silicone Co., Ltd. (Cixi, China). The silane coupling agent was triethoxyvinylsilane, and its molecular weight was 190 g/mol, purchased from Shouzheng Chemical Technology Co., Ltd. (Guangzhou, China). The manufacturing process of silicone rubber included the following four steps. First, a methyl-terminated PDMS and the hydride-functional silicone oils were fully mixed at 50 ${}^{\circ }$C for 5 h. During this mixing process, some necessary fillers, such as aluminium hydroxide, zinc oxide, and amorphous silica, were also added. Second, after the mixture cooled down, we added the 2,5-dimethyl-2,5-di (tert-butylperoxy) hexane catalyst into the mixture by open mixing in double-roller blending rolls for 10 min. Third, the mixture obtained from the first two steps was vulcanized in a mould at 170 ${}^{\circ }$C, 15 Mpa for 10 min. Fourth, we put the vulcanized silicone rubber in an electric blast drying oven for the post-curing process at 170 ${}^{\circ }$C for 6 h.

### 2.2. Ultraviolet Irradiation

Before irradiation, samples were cut into a plate with a dimension of 20 × 20 × 1 mm${}^{3}$. The irradiations were performed by the Q-lab’s commercial UVA-340 nm (center wavelength 340 nm) and UVB-313 nm lamps (center wavelength 310 nm), respectively. Only one side of the sample was exposed to ultraviolet rays. The distance between the UVA/UVB source and the PDMS composite was 50 mm. The UVA/UVB radiation power density was 55 mW/cm${}^{2}$, and the temperature of the radiation chamber was regulated at 50 ${}^{\circ }$C. All the samples were exposed to UVA/UVB irradiation for different periods of time.

### 2.3. Extraction of the Uncrosslinked Siloxanes

The uncrosslinked LMW siloxanes in silicone rubber were extracted in a soxhlet apparatus. A total weight of 25 g of sample was cut into pieces for extraction. The solvent was n-hexane, analytical regent grade, for which the heating temperature was 90 ${}^{\circ }$C. The amount of used n-hexane was approximately 200 mL, which was able to immerse all the silicone rubber samples in the soxhlet apparatus. Based on our previous study, we found that 96 h of heating and reflux could extract all the LMW siloxanes in the bulk of a silicone rubber. A detailed discussion can be found in the Supporting Information. After 96 h of extraction, the extract solution was concentrated to 10 mL using a rotatory evaporator under 90 ${}^{\circ }$C. The purpose of using rotatory evaporation was to concentrate the extracts for quantitative analysis. A higher concentration of the silicone oligomers solution makes it easier for the GC–MS to detect, because the instrument has a detection limit.

### 2.4. GC–MS and GC Analysis

GC–MS was mainly used to identify the structure of the LMW siloxanes in the silicone rubber extracts. The instruments used were the Varian 450-gas chromatography 320-mass spectrometry, coupled with a thermal conductivity detector (TCD). The separation was carried out in a Varian VF-5ms capillary column. Helium was used as the carrier gas. The injector port temperature was set at 280 ${}^{\circ }$C. The initial oven temperature was 50 ${}^{\circ }$C. This temperature was kept for 3 min, after which the temperature was linearly increased to 290 ${}^{\circ }$C at a rate of 20 ${}^{\circ }$C/min. The final temperature was held for another 10 min. The TCD detector temperature was set at 280 ${}^{\circ }$C. The mass spectrometer was operated at 70 eV. The transfer line between GC and MS was 260 ${}^{\circ }$C. The mass spectra of LMW species were verified by comparison of the identified species with the NIST08 database.

GC was used to quantify the extracted LMW siloxanes, using an external standard method. The instrument was an Agilent 6890 gas chromatography, equipped with a flame ionization detector (FID). The injector port temperature and the heating program were set to the same as the GC–MS. Octamethyl cyclotetrasiloxane (D${}_{4}$) was used as the standard substance. A linear relationship between D${}_{4}$ concentration and its peak area was determined. The calibration was based on the assumption that the D${}_{4}$ calibration curves were also valid for other LMW siloxanes.

### 2.5. Positron Annihilation Lifetime Spectroscopy

Positron annihilation lifetime spectroscopy (PALS) was used to probe the free volume changes in silicone rubber after UVB irradiation. Before PALS measurement, each sample was extracted in a soxhlet apparatus using n-hexane. In order to remove the solvent, n-hexane, the extracted samples were stored in a vacuum chamber at 5 Pa for 24 h. Then, the silicone rubbers were cut into dimensions of 20 × 20 × 1 mm${}^{3}$ for PALS measurement. The PALS data were collected using a conventional fast–fast coincidence system with a time resolution of 300 ps. A ${}^{22}$Na positron source was sandwiched between two pieces of silicone rubber. The measurement was conducted at room temperature and each spectrum had a total count of one million. Each obtained lifetime spectroscopy was analyzed by the PATFIT program [17]. Other details of the experiment can be found in our published works [18].

## 3. Results and Discussion

### 3.1. Extracts in Silicone Rubber Identified by GC–MS

**Figure 1:**
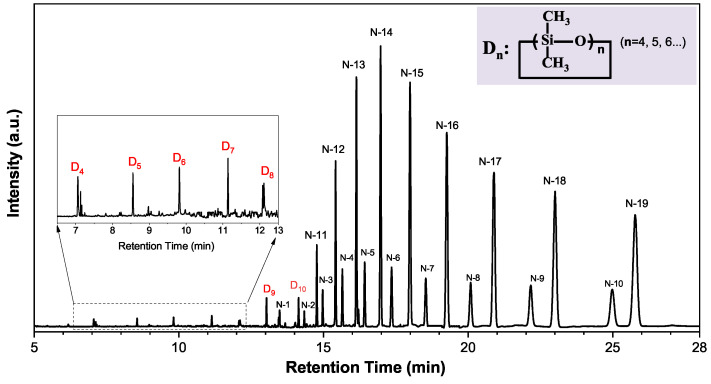
Gas chromatogram showing the homologous peak marks of the extracts in virgin silicone rubber.

In GC–MS, the function of GC is to separate the substances, while the mass spectrometry is to identify the corresponding structure of the substance. As shown in Figure 1, each peak in GC has a corresponding mass spectrum, so each of them represents a kind of extracted LMW silicone. Two methods were used to identify the structure of each extracted LMW silicone by mass spectra: first, through automatic matching with the standard cards in the NIST08 database in the GC–MS software and second, through manual analysis of the mass spectrum.

In Figure 1, the peaks marked from D${}_{4}$ to D${}_{10}$ are identified by comparing with the NIST08 data base. The result indicates that the extracts are mainly siloxanes. Lager retention time peaks, from N-1 to N-19, cannot be identified by this method because their mass spectrum peaks are similar, resulting in a rather low comparing similarity with the standard card. As a result, the structures of the peaks from N-1 to N-19 were identified by the manual analysis.

**Figure 2:**
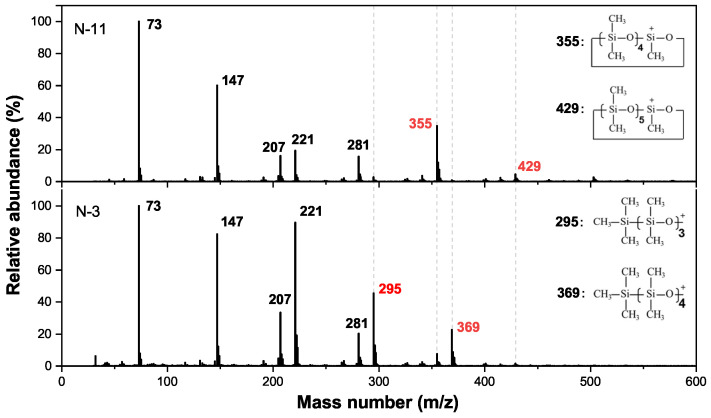
Mass spectra of N-3 and N-11 in GC–MS of the silicone rubber extracts.

In mass spectra, unknown peaks from N-1 to N-19 all contain *m/z* of 73, 147, 207, 221, and 281, as shown in Figure 2, where *m* is the mean atomic mass and *z* is the charge of the detected ion. Here, the *m/z* of 73, 147 and 221 correspond to the structure of [+Si(CH${}_{3}$)${}_{3}$], [+(CH${}_{3}$)${}_{2}$SiOSi(CH${}_{3}$)${}_{3}$], and [+((CH${}_{3}$)${}_{2}$SiO)${}_{2}$Si(CH${}_{3}$)${}_{3}$], which originate from the methyl shift process after the electron impact. By contrast, the *m/z* of 207, 281 results from the loss of a methyl group (CH${}_{3}$) in D${}_{3}$ and D${}_{4}$, respectively [19]. The results indicate that peaks from N-1 to N-19 are also siloxanes, judging by the distinctive mass number, that is, *m/z* of 73, 147, 207, 221 and 281. Therefore, we propose that successive peaks in Figure 1 correspond to a unit increase in the number of dimethylsiloxane units.

As shown in Figure 2, structures for *m/z* of 295, 355, 369, and 429 are plotted. It can be seen that the *m/z* of 355 and 429 are fragments of cyclic siloxanes, while the *m/z* of 29 and 369 are fragments of linear siloxanes. The differences between the mass spectra of N-3 and N-11 are that N-3 has more *m/z* of 295, 369 than N-11, and N-11 has more *m/z* of 355, 429 than N-3. The result indicates that N-3 has many more methyl end-groups. Referring to the data on soft ionization published in the literature [20], we propose that peak N-3 is a linear LMW siloxane, and peak N-11 is a cyclic LMW siloxane. Thus, the peak N-11 represents the substance of D${}_{11}$, while N-3 represents the substance of L${}_{10}$ (L${}_{n}$=CH${}_{3}$[(CH${}_{3}$)${}_{2}$SiO]${}_{n}$Si(CH${}_{3}$)${}_{3}$). Based on the above discussion, peaks from N-11 to N-19 are cyclic siloxanes from D${}_{11}$ to D${}_{19}$, and peaks from N-1 to N-10 are linear siloxanes from L${}_{8}$ to L${}_{17}$.

### 3.2. Quantitative Analysis of Extracts in Silicone Rubber Irradiated by Sunlight UV

**Figure 3:**
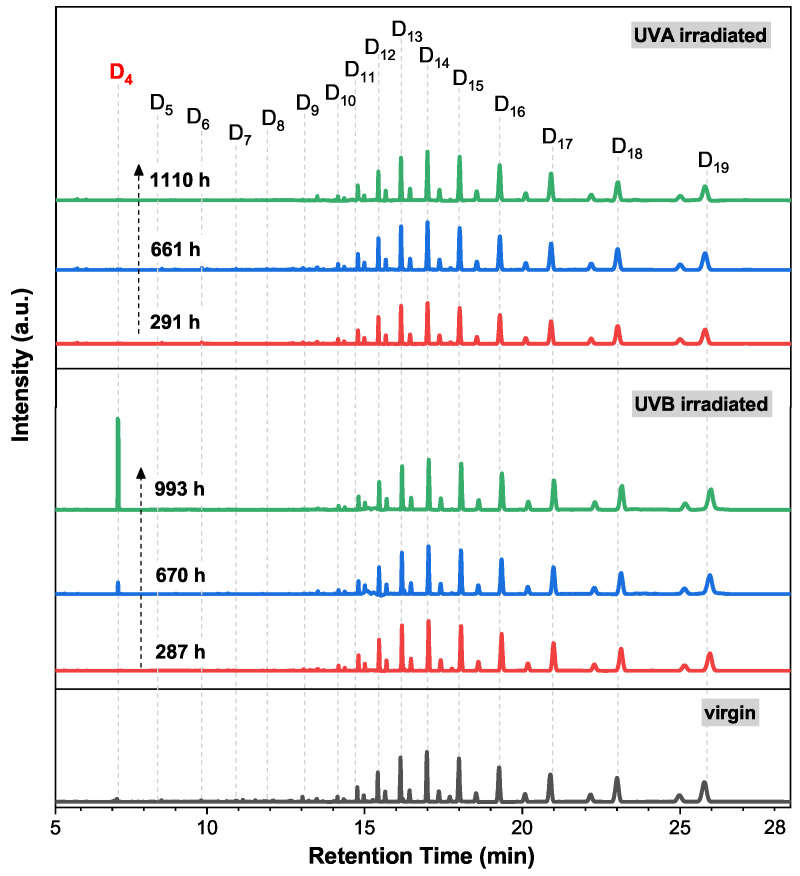
GC peak variations of the silicone rubber extracts after different durations of UVA and UVB irradiation.

An overall variation trend of LMW siloxanes in silicone rubber after UVA/UVB irradiation is shown in Figure 3. Each peak’s intensity in GC remains almost unchanged after UVA irradiation, while the intensity of peak D${}_{4}$ grows after UVB irradiation. This result indicates that the PDMS networks in silicone rubber are stable in UVA light, and this also corresponds with the results reported in the literature [13,14]. However, after UVB irradiation, the PDMS network in silicone rubber would decompose. This result is related to the photon energy differences between UVA and UVB. The wavelength range of UVB (280∼340 nm) is much shorter than UVA (300∼380 nm), and thus the maximum photon energy of UVB (4.44 eV) is much higher than that of UVA (4.14 eV). In our previous study, we found that UVB light could break the Si-C bond in PDMS and lead to the generation of a silica-like layer on a silicone rubber surface. In this study, it was found that UVB light could also cause the generation of D${}_{4}$ in the bulk of a silicone rubber.

**Figure 4:**
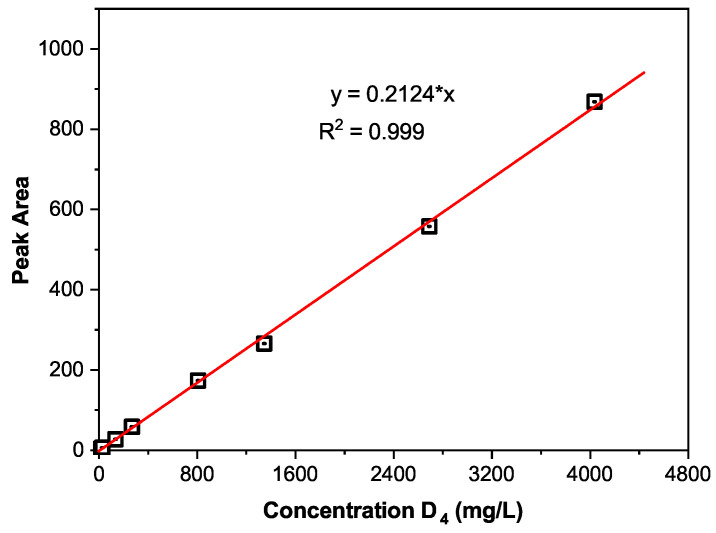
The calibration curve for D${}_{4}$ in n-hexane: GC peak area as a function of D${}_{4}$ concentration.

In order to conduct the quantitative analysis, the calibration curve of D${}_{4}$ was obtained, as shown in Figure 4. The value of the correlation coefficients (R${}^{2}$) is 0.999, very close to 1.0, indicating good linearity of the detector within the considered concentration range. In Figure 4, the obtained linear function between the D${}_{4}$ concentration and its peak area in n-hexane is *y* = 0.2124*x, where *y* is the peak area, *x* is the D${}_{4}$ concentration. The formula used to calculate the concentrations of the extracted LMW is:

(1) $$C_{\mathrm{LMW}}=S_{\mathrm{LMW}}/k,$$

where *C*${}_{\mathrm{LMW}}$ is the concentration for one kind of LMW, *S*${}_{\mathrm{LMW}}$ is the peak area of the LMW peak in GC, *k* is the parameter obtained by the D${}_{4}$ calibration, which is 0.2124 mg/L${}^{-1}$ in our study.

**Figure 5:**
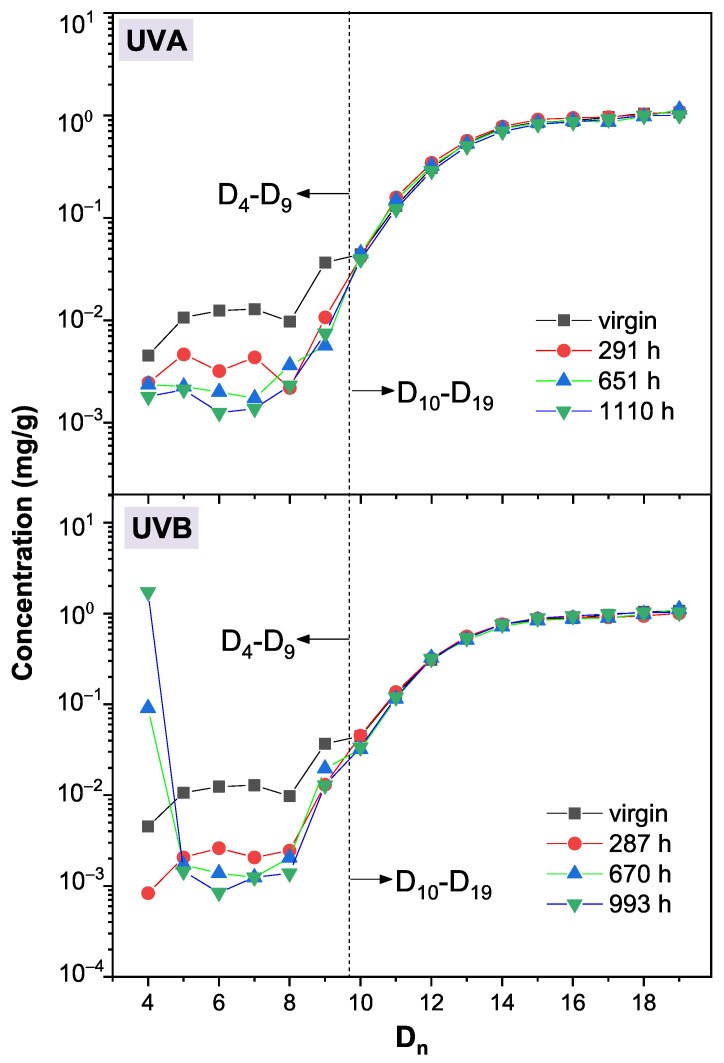
The calculated concentration of cyclic LMW siloxanes in virgin and exposed samples: UVA exposed (**upper**); UVB exposed (**below**).

Using Equation (1) for further calculation, as shown in Figure 5 (upper), the concentrations of cyclic siloxanes with a low repeat unit (D${}_{4}$–D${}_{9}$) decrease after UVA irradiation, while the concentrations of the larger cyclic siloxanes (D${}_{10}$–D${}_{19}$) remain almost unchanged. The variations of L${}_{n}$ are shown in Figure S3, showing that L${}_{n}$ also remains unchanged. Here, mg/g is a unit that describes the content of the oligomer (mg) in each gram of silicone rubber. Combined with the data obtained by PALS, we propose that D${}_{4}$–D${}_{9}$ are crosslinked to the PDMS network in silicone rubber, which is discussed in the PALS part of this paper. For a cyclic siloxane, its stability is usually related to its ring stain, and a lower repeat unit results in a higher ring stain. Since the high ring strain is more likely to lead to the breaking of chemical bonds, the D${}_{4}$–D${}_{9}$ will be more reactive than those with a high repeat unit in regular conditions. Moreover, these siloxanes with a low repeat unit (D${}_{4}$–D${}_{9}$) could diffuse freely in the bulk of silicone rubber. This contributes to a higher possibility of taking part in the crosslinking reaction, which could be catalyzed by the impurity ions in the fillers. As a result, D${}_{4}$–D${}_{9}$ are more likely to crosslink than D${}_{10}$–D${}_{19}$, resulting in a lower concentration of D${}_{4}$–D${}_{9}$ after UVA/UVB irradiation.

**Figure 6:**
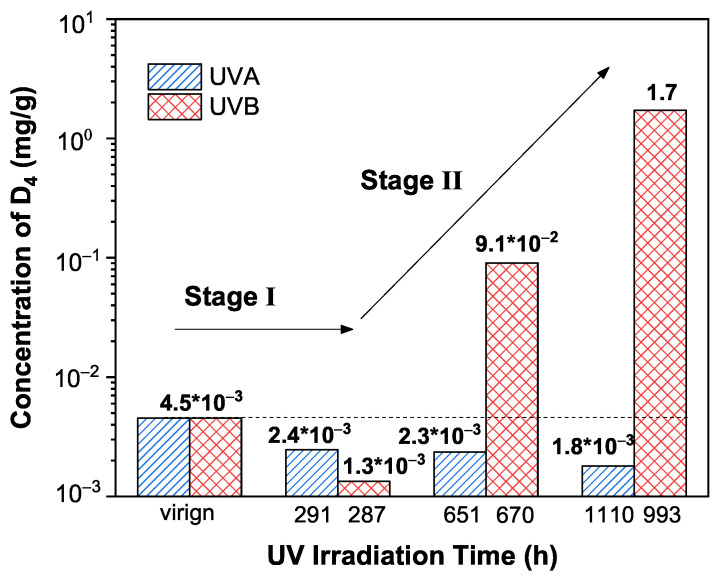
The calculated concentration of D${}_{4}$ in virgin and UVA/UVB irradiated samples.

After 287 h of UVB irradiation, as shown in Figure 5 (below), the concentration of D${}_{4}$-D${}_{9}$ also decreases, suggesting a crosslinking process of these silicone oligomers. However, as the UVB irradiation time increases to 993 h, only the concentration of D${}_{4}$ increases. Since the concentrations of other oligomers (D${}_{5}$–D${}_{20}$) remain almost unchanged, we deduce that the newly generated D${}_{4}$ originates from the decomposition of the PDMS network in silicone rubber, rather than those oligomers larger than D${}_{4}$. Hence, the concentration of D${}_{4}$ can be used as a feature parameter to evaluate the degradation in the PDMS network of silicone rubber after UVB irradiation. As shown in Figure 6, the concentrations of D${}_{4}$ as a function of irradiation time are plotted. The concentration of D${}_{4}$ in virgin silicone rubber is 4.5 × 10${}^{-3}$ mg/g. After 291 h of UVA irradiation, the concentration of D${}_{4}$ decreases to 2.4 × 10${}^{-3}$ mg/g. This decrement of D${}_{4}$ has also been found during the first 287 h of UVB irradiation. As mentioned above, this decrement of D${}_{4}$ indicates a UVA/UVB irradiation induced crosslinking process in silicone rubber. The same crosslinking process has also been found in other polymers during the first few hundred hours of UV irradiation [21], which is termed the “post curing” process (Stage I). After 651 h or 1110 h of UVA irradiation, the concentration of D${}_{4}$ in silicone rubber is unchanged, remaining at approximately 2.0 × 10${}^{-3}$ mg/g. By contrast, longer durations (670, 993 h) of UVB irradiation cause the generation of D${}_{4}$ in silicone rubber. This is the newly found aging behavior of silicone rubber under UVB irradiation, and we call it the “chain scission” process (Stage II). Based on the above discussion, the differences between the impacts of the UVA and UVB irradiation on the aging of silicone rubber are obtained: UVA irradiation only causes a post curing process; UVB irradiation could cause chain scission in silicone rubber. The generated D${}_{4}$ also facilitates the hydrophobic recovery process of silicone rubber, which is discussed in Figure S5 (in the Supporting Information).

### 3.3. Free Volumes Characterized by PALS

The PALS was used to probe the free volume changes in silicone rubber after UVB irradiation. Free volume is the space that is not occupied in a polymer [22]. The variation of free volume is usually related to the crosslinking state of the polymer network [23,24]. A positron is the antiparticle of an electron, and it will annihilate with an electron. When a positron penetrates into a polymer, it might directly annihilate with an electron, or capture an electron to form a positronium, a hydrogen-like bound state of positron and electron, and then annihilate. Direct annihilation of a positron and an electron is called free positron annihilation, the duration of which is approximately 300 ps. By contrast, a positronium has two kinds of annihilations due to its two kinds of spin states. One is the spin antiparallel state, which results in the para-positronium (p-Ps) annihilation with a lifetime of about 125 ps; the other is the spin parallel state, which results in the ortho-positronium (o-Ps) annihilation [25,26]. The lifetime of o-Ps is 142 ns in a vacuum, but if it is formed in a polymer, its lifetime would be shortened to 1–5 ns, due to the so-called “pick-off” annihilation [27,28]. The lifetimes of p-Ps, a free positron, and o-Ps are usually recorded by $\tau_{1}$, $\tau_{2}$, and $\tau_{3}$, respectively. Here, only the o-Ps annihilation is related to the free volume information in a polymer. For some polymers containing a rich free volume, the o-Ps would annihilate in more than one kind of free volume. In order to obtain a better fitting variance in PATFIT, a fitting of four lifetimes would be used to analyze the PALS spectrum. In this paper, $\tau_{3}$ and $\tau_{4}$ are both contributed by the o-Ps annihilation. According to our previous study, $\tau_{3}$ is ascribed to the o-Ps annihilation in free volumes that exist near the PDMS crosslinking site, while $\tau_{4}$ is ascribed to the o-Ps annihilation in the free volumes between the clusters of the network [18,29].

**Figure 7:**
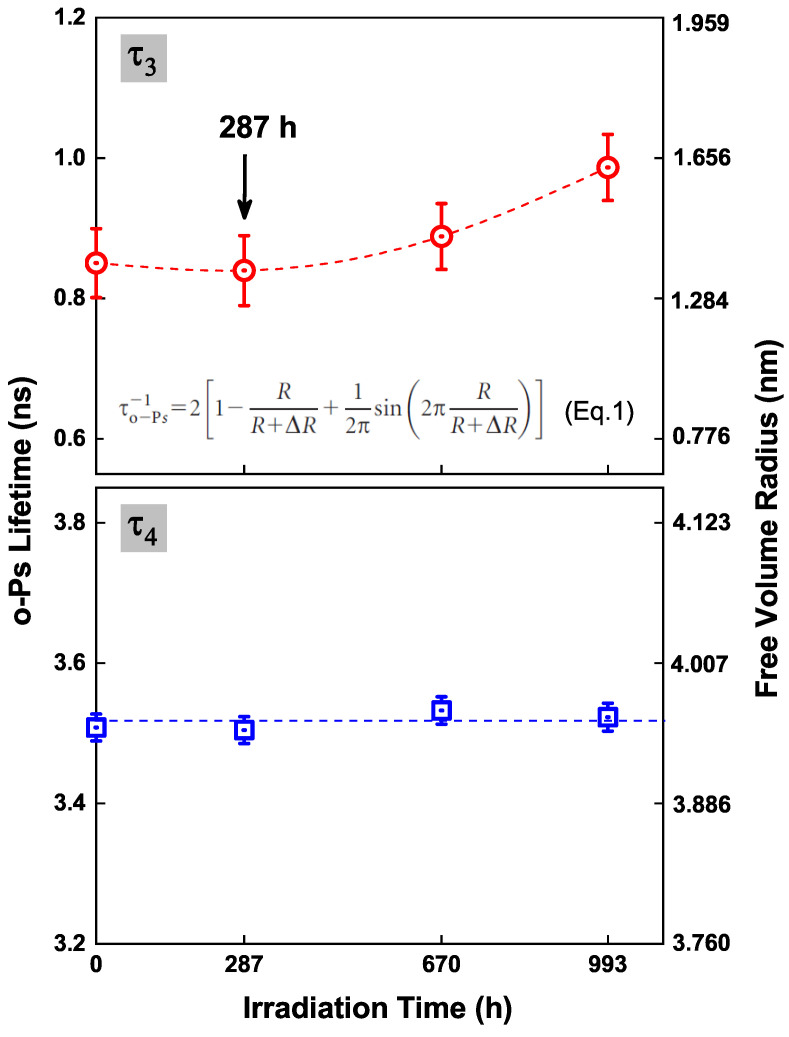
The o-Ps lifetimes ($\tau_{3}$, $\tau_{4}$) and the corresponding free volume sizes (according to Tao’s model [30]) in silicone rubbers after different durations of UVB irradiation.

As shown in Figure 7, the value of $\tau_{3}$ remains at approximately 0.85 ns after the first 287 h of UVB irradiation, and then increases to 1.0 ns as the UVB irradiation time extends to 993 h. The increase of $\tau_{3}$ indicates the growing size of free volumes near the PDMS crosslinking sites. According to the data obtained by GC, UVB irradiation could result in the chain session of PDMS and the generation of D${}_{4}$ in silicone rubber. As the crosslinked PDMS network decoposes in bulk of silicone rubber, the size of free volumes near the crosslinking sites will increse, thus resuling in the increment of $\tau_{3}$. By contrast, the value of $\tau_{4}$ remains almost unchanged during the 993 h of UVB irradiation. The result indicates that, UVB irradiation dose induce chain scissions in a silicone rubber, but affects little in the size of the bigger free volumes localized in the clusters of the PDMS network, suggesting a relatively high-elastic state of the irradiated silicone rubber.

**Figure 8:**
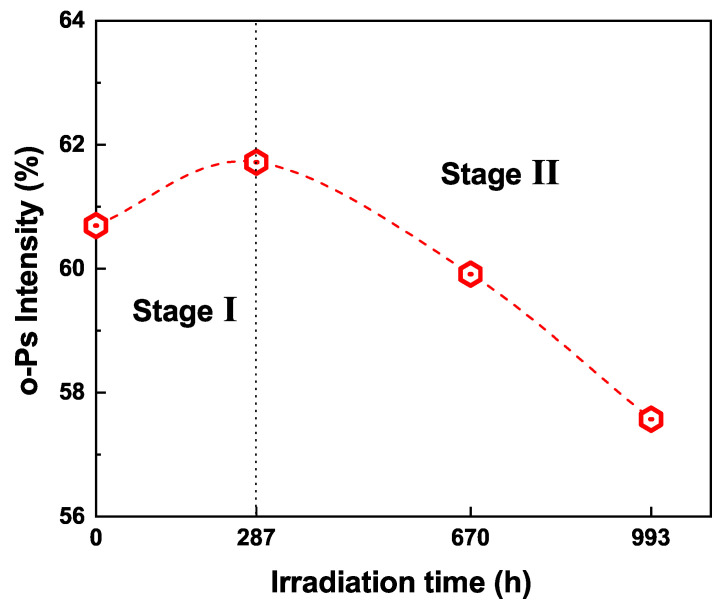
The o-Ps intensity of silicone rubber after different durations of UVB irradiation.

The variation of o-Ps intensity is shown in Figure 8. The o-Ps intensity is the sum intensity of $\tau_{3}$ and $\tau_{4}$, reflecting the total amount of free volumes in the sample. The o-Ps intensity first increases slightly from 60.6% to 61.7% during the first 287 h of UVB irradiation, and then starts to decrease, reaching 57.5% as the UVB irradiation time is prolonged to 993 h. It has already been proven that o-Ps does not form in the ATH—the most used inorganic filler in silicone rubber [18,31]. The o-Ps usually forms in the organic component in a polymeric composite. In this way, we propose that the o-Ps intensity is mainly determined by the relative content of organic PDMS in silicone rubber. It should be mentioned that the organic PDMS has two existing states: one is the crosslinked PDMS network; the other is the uncrosslinked PDMS oligomers. In our study, the uncrosslinked PDMS oligomers had already been removed by an extraction process before the PALS measurement, hence the o-Ps intensity only reflects the relative content of the crosslinked PDMS network in silicone rubber. The increase of the o-Ps intensity in the first 287 h of UVB irradiation is due to the crosslinking of the uncrosslinked PDMS oligomers in the “post curing" process, which results in the increased content of the crosslinked PDMS. The decrease of the o-Ps intensity is due to the decomposition of the PDMS network into D${}_{4}$ in the “chain scission" process, which leads to the decomposition of the PDMS network into the uncrosslinked oligomers. The PALS result corresponds well with the GC result, and it provides new evidence for the UVB induced post curing and chain scission processes in silicone rubber.

### 3.4. Aging Mechanism of Silicone Rubber under UVA/UVB Irradiation

Two aging processes in silicone rubber under UVA/UVB irradiation are obtained: the first is the post curing process; the second is the chain scission process. The former usually occurs after the first few hundred hours of irradiation, and has already been found after UVA and UVB irradiation. The latter occurs after a long duration (>300 h) of UVB irradiation, and only $\tau_{4}$ is generated. Since the average photon energy of UVB (428 kJ/mol) is much higher than that of UVA (399 kJ/mol), photons of UVB can initiate the rupture of chemical bonds more effectively. The bond energies of C-H, Si-C and Si-O in PDMS are 414 kJ/mol, 301 kJ/mol, and 447 kJ/mol, respectively [32]. The photon energy of UVB is enough to break the C-H and Si-C bond, but not enough to break the Si-O bond. This indicates that the UVB induced rupture of the Si-O bond is not determined by the photon energy.

For the UVB induced PDMS decomposition found in this study, we propose that the rupture of the C-H bond is important for the aging process. The C-H bond is stable with UVA, and it reflects UVA light like an umbrella from the outward structure of PDMS, so the Si-C or Si-O can hardly be influenced by the UVA light. By contrast, the C-H bond will break under the UVB irradiation, and then the Si-C bond can also be broken, resulting in the generation of free radicals that contain silicon atoms. These unstable free radicals will further react with ambient water molecules to generate the silanol groups. According to the studies reported in the literature, a silanol group at the end of the PDMS chain could trigger a so-called “end-initial” decomposition of PDMS [11,33]. It is worth noting that only D${}_{4}$ is generated in this process. The UVB induced backbiting decomposition tendency of PDMS might be related to the chemical environment provided by the fillers in silicone rubber. The fact that D${}_{4}$ is generated after a long duration (>300 h) of UVB irradiation is the newly found degradation behavior of silicone rubber in this study. This finding can be used to quantitatively evaluate the UVB stability of silicone rubber by way of GC, and also provides new insight for optimizing the insulation strategy in UV-rich plateau places.

## 4. Conclusions

In this paper, it is found that silicone rubber undergoes two aging processes under UVA/UVB irradiation: the post curing process and the chain scission process. The post curing process occurs after the first few hundred hours (<300 h) of irradiation. In this process, the uncrosslinked cyclic oligomers, D${}_{4}$–D${}_{9}$, are crosslinked into the PDMS network. The chain scission process only occurs after the long duration (>300 h) of UVB irradiation. The principal feature of this aging process is the generation of only D${}_{4}$. This difference is ascribed to the fact that the photon energy of UVB is higher than that of UVA. UVB photons could break the C-H bond and trigger the backbiting decomposition of PDMS, while UVA photons cannot. The finding that only D${}_{4}$ was generated after UVB irradiation can be used to quantitatively evaluate silicone rubber’s UVB resistance.

## Figures and Tables

**Table 1:** The content of the raw materials used in the silicone rubber.

Raw Material Type	Raw Material Name	Weight	Percentage
		(phr)	(wt.%)
organic component	methyl-terminated PDMS	100	46.51
inorganic fillers	aluminum hydroxide (ATH)	70	32.56
	amorphous silica (SiO${}_{2}$)	25	11.63
	zinc oxide (ZnO)	2	0.93
assistant additives	hydride-functional silicone oil	10	2.79
	silane coupling agent	6	4.65
	2,5-dimethyl-2,5-di(tert-butylperoxy)	2	0.93

## Data Availability

The data presented in this study are available on request from the corresponding author. The data are not publicly available due to privacy.

## Supplementary Materials

The supplementary materials are available online at https://www.mdpi.com/article/10.3390/polym13132215/s1—Figure s1: Natural summer noon sunlight in the UV region compared with emission spectra of UVA-340 and UVb-313 light sources of the Q-lab’s product, Figure s2: Weight loss of silicone rubber as a function of extraction time, Figure S3: Photos of the silicone rubber before and after UVA/UVB irradiation, Figure S4 The calculated concentration of linear LMW siloxanes in virgin and UV exposed samples: UVA exposed (upper); UVB exposed (below), Figure S5: The hydrophobic recovery curves of silicone rubber after different times of UVB irradiation, (a) contact angle verse recovery time; (b) contact angle verse log(t).
