# Supplementary material for: New Aspects of Degradation in Silicone Rubber under UVA and UVB Irradiation: A Gas Chromatography–Mass Spectrometry Study
Source: Polymers (Basel). 2021 Jul 5;13(13):2215. doi: 10.3390/polym13132215 (PMC8271979; doi:10.3390/polym13132215)
Supplement: Supplementary file 1 [file polymers-13-02215-s001.zip › polymers-1209729-SI.pdf]

polymers

Supporting Information

New Aspects of Degradation in Silicone Rubber under UVA and UVB Irradiation: A Gas Chromatography-Mass Spectrometry Study

Zheng Wang [1,2], Libing Qian [2], Xiangyang Peng [1,*], Zhen Huang [1], Yue Yang [2] and Chunqing He [2,*] and Pengfei Fang [2,*]

[1] Department of Physics and Hubei Nuclear-Solid Physics Key Laboratory, Wuhan University, Wuhan 430072, China
[2] Guangdong Key Laboratory of Electric Power Equipment Reliability, Electric Power Research Institute of Guangdong Power Grid Co.,Ltd, Guangzhou 510080, Chin
* Correspondence: pigpxy@126.com (X.P.); hecq@whu.edu.cn (C.H.); fangpf@whu.edu.cn (P.F.)
Tel.: +86-020-85123465 (X.P.); fax: +86-020-85123465 (X.P.)

1. Experiments

1.1. UVA/UVB irradiation

Two types of Q-lab's commercial UVA/UVB light sources were used: UVA-340 nm, UVB-313 nm. The spectra of sunlight in the UV region, UVA-340 nm, and UVB-313 nm are plotted in Figure S1. The highest intensity of UVA-340 nm spectrum is at 340 nm, while UVB-313 nm is at 313 nm.

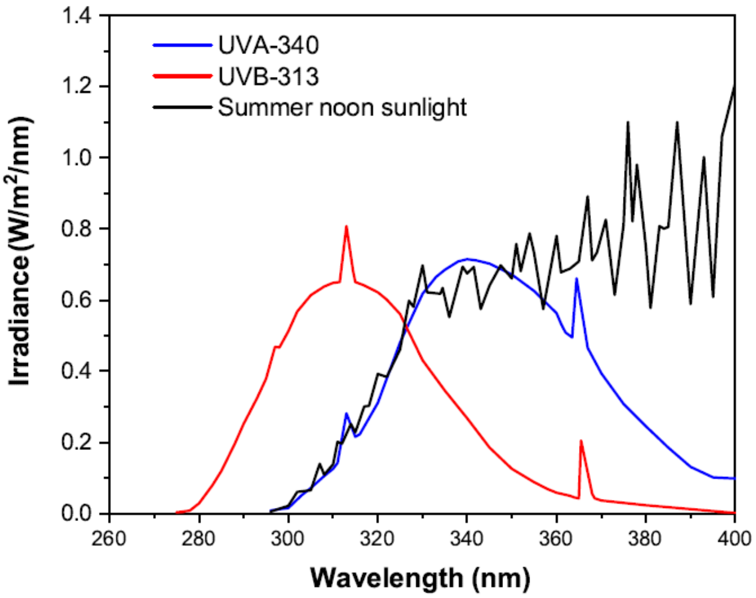

Figure S1. Natural summer noon sunlight in the UV region compared with emission spectra of UVA-340 and UVb-313 light sources of the Q-lab's product.

1.2. Extraction time of the uncrosslinked siloxanes

In order to extracted all the uncrosslinked siloxanes in silicone rubber, a 96 h extraction was used. This 96 h extraction is based on our previous study. The weight loss of silicone rubber as a function of its extraction time is shown in Figure S2. The relative weight loss of silicone rubber remains unchanged after at least 96 h of extraction, indicating all the oligomers in the bulk of silicone rubber are extracted. The final weight of the

silicone rubber was obtained by a multiple weighing method. Before each weight measurement, the sample was stored in the vacuum drying oven for 12 h, at 5000 Pa, 50 ℃.

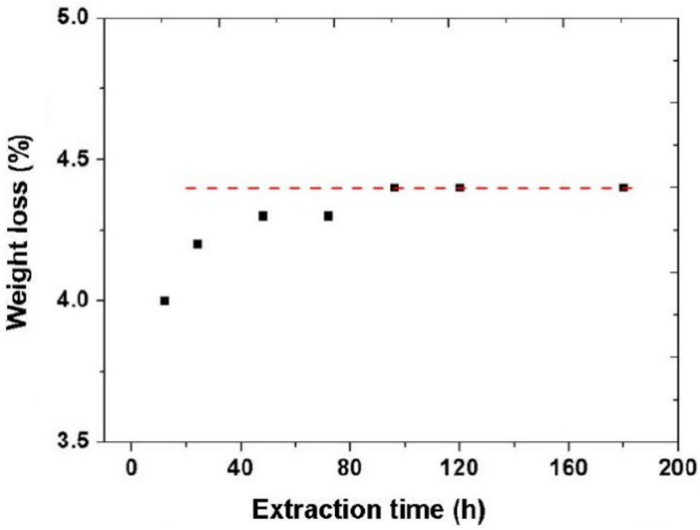

Figure S2. Weight loss of silicone rubber as a function of extraction time.

1.3. Plasma treatment and hydrophobic recovery measurement

Plasma treatments were performed using a 13.56 MHz radio frequency (RF) plasma reactor (YZD08-2A) purchased from Tangshan Yanzhao technique institute, China, of which working power was 80 W. The UV aged side of the sample was used for plasma treatment and hydrophobic recovery measurement. Argon was used as the reacting gas, since argon plasma will not introduce new element on sample surface during the treatment. The pressure in chamber was controlled approximately 20 Pa. The whole plasma treatment process lasted for 3 min. After plasma treatment, each sample was kept in a chamber, of which humidity and temperature were regulated at 50 %, 23 ℃, respectively.

Hydrophobic recovery was evaluated by static contact angle of the sample after plasma treatment. Static contact angle was measured to evaluate surface hydrophobicity of each sample using a goniometer (CAST3, Kino, USA) to an accuracy of $\pm 3\theta$. Each single droplet of deionized water was controlled in a volume of about 10 μL while it was applied on the surface of PDMS composite. Five locations were examined each time contact angle values were recorded.

2. Results and discussion

2.1. Sample appearance

The photos of the silicone rubber before and after UVA/UVB irradiation are shown in Figure S3. The appearance of the UVA/UVB aged samples are the same with the virgin ones. No differences could be found just by the photos of the samples. Hence, it is impossible to judge the aging degree by the sample appearance.

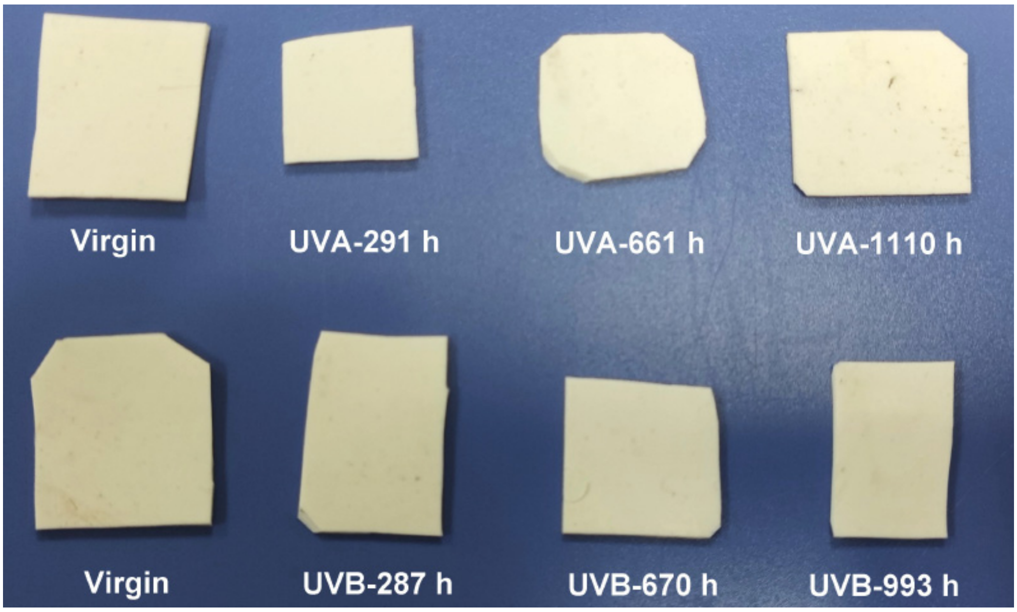

Figure S3. Photos of the silicone rubber before and after UVA/UVB irradiation.

2.2. Plasma exposure and hydrophobic recovery measurements

As shown in Figure S4, the concentration of linear siloxanes (Ln=CH3[(CH3)2SiO]nSi(CH3)3) show no systematic change with UVA or UVB exposure time. The result indicates that, after different times of UVA or UVB irradiations, no linear siloxanes were generated.

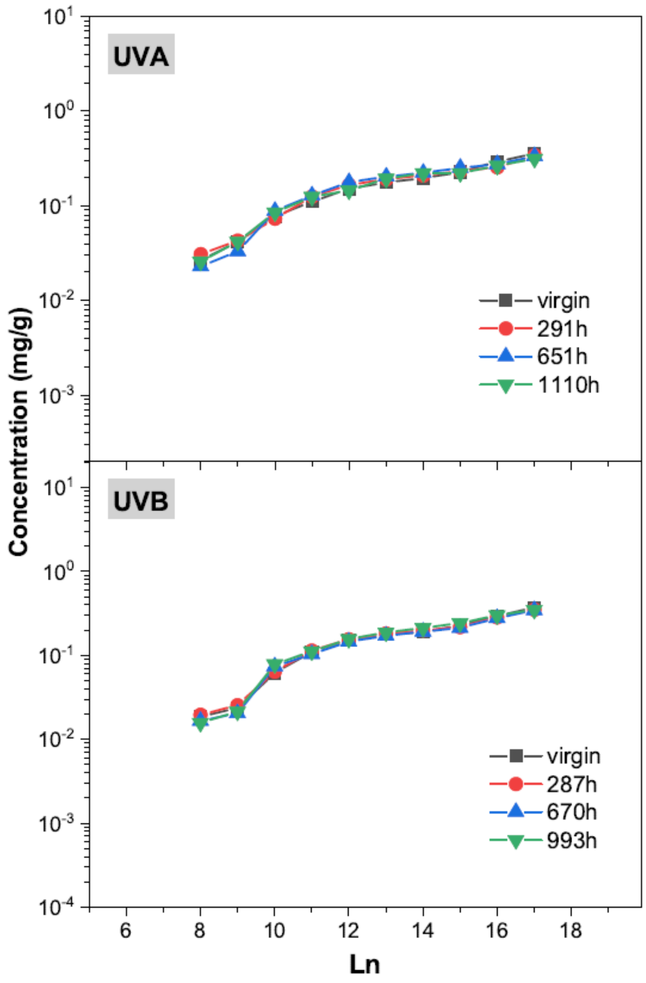

Figure S4. The calculated concentration of linear LMW siloxanes in virgin and UV exposed samples: UVA exposed (upper); UVB exposed (below).

2.3. Plasma exposure and hydrophobic recovery measurements

As shown in Figure S5(a), each sample surface turns into totally hydrophilic (0) after 3 min of plasma treatment. After a few hours of recovery, contact angle of each sample increases quickly. To get more useful information on the early time of hydrophobic recovery, we convert the x axis from time into logarithm of time in Figure S5(b). A linear fitting was used to quantitatively analyze the hydrophobic recovery rate of each sample. Here, k is the slope of the fitted line, and it is directly proportional to the recovery rate. The k values of the virgin sample and the 287 h-aged one are in the same level, equaling approximately 55. By contrast, the k values of the 670 h-aged and the 993 h-aged samples have an increasing trend, equaling 60.2 and 65.7, respectively. The hydrophobic recovery of silicone rubber is related to the amount of LMW siloxanes in its bulk. Since the long time (> 670 h) aged samples have more D_4 in its bulk according to the GC-MS result, these two samples can recover more quickly.

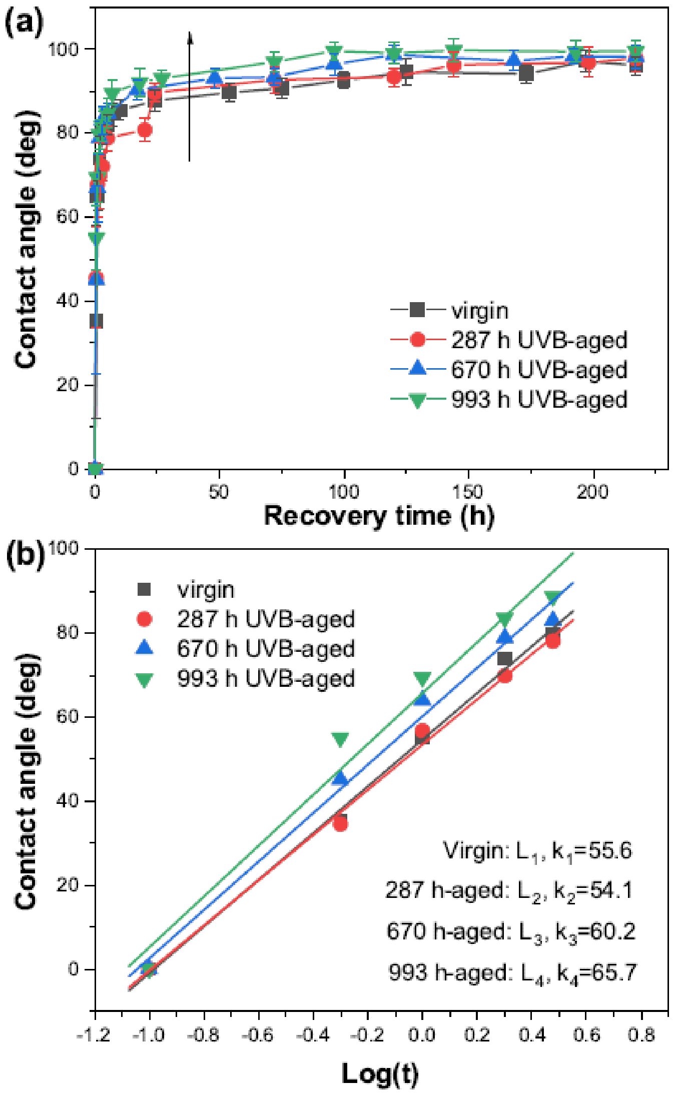

Figure S5. The hydrophobic recovery curves of silicone rubber after different times of UVB irradiation, (a) contact angle verse recovery time; (b) contact angle verse log(t).
